# High Performance (High Pressure) Layer Electrochromatography Separation Technique: Equipment and Preliminary Results

**DOI:** 10.1021/acs.analchem.2c01376

**Published:** 2022-06-14

**Authors:** Radosław Łukasz Gwarda, Tadeusz Henryk Dzido

**Affiliations:** Department of Physical Chemistry, Chair of Chemistry, Faculty of Pharmacy, Medical University of Lublin, 4a Chodźki Str., 20-093 Lublin, Poland

## Abstract

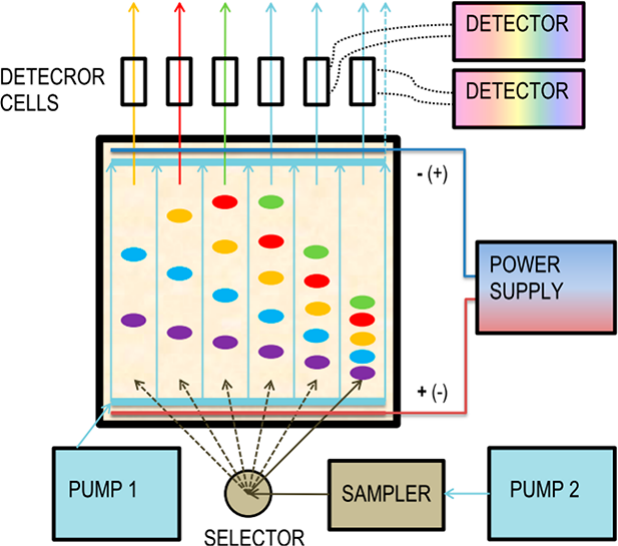

We present a new prototype device and propose a new analytical technique: high performance (high pressure) layer electrochromatography (HPLEC). The equipment provides a combination of overpressured layer chromatography (OPLC) and pressurized planar electrochromatography (PPEC), yet it still enables researchers to perform each of these analyses separately. In comparison to PPEC, HPLEC provides hydrodynamic flow of the mobile phase, irrespective of the voltage used and the mobile phase composition. The advantages of HPLEC over OPLC include the possibility of the use of the electrophoretic effect to influence the selectivity of separation and the use of the electroosmotic effect to facilitate the mobile phase flow in order to decrease backpressure and increase the flow velocity. Many operational parameters can be freely adjusted and optimized independently. The equipment is fully automated and can work in various separation/operational modes, including combinations of online/offline sample application and detection. We present preliminary results of simultaneous, fully online, multichannel HPLEC separation of analgesic drugs (including acetaminophen, ibuprofen, and tramadol) as an example of increasing analysis throughput.

Among many various liquid chromatography (LC) techniques established so far, two main variants concerning the form/shape of the adsorbent bed can be distinguished: column liquid chromatography (CLC) and layer liquid chromatography (LLC).^1−3^ The most widespread technique used in the laboratory practice is CLC, especially high-performance liquid chromatography (HPLC), that is, a version of CLC with the mobile phase flow driven by high external pressure applied to the column inlet. This results from the fact that the technical conditions make forcing a rapid and uniform eluent flow through the chromatographic column relatively easy. Therefore, both scaling the adsorbent bed (e.g., changing the column length) and increasing the separation performance (decreasing the analysis time) are also relatively easy.^2,4−6^

On the contrary, LLC is characterized by other important advantages, that is, the possibility of the separation of multiple samples at the same time or the wide options of solute derivatization and detection. However, due to technical issues, forcing a rapid and uniform flow of the mobile phase through the adsorbent layer is much more difficult and requires additional technical solutions. Hence, the very simple variant, that is, thin-layer chromatography (TLC; and a high-performance version of TLC (HPTLC)) remains the most common LLC technique used in laboratories. Still, its performance is lower in comparison to HPLC. In TLC, the flow of the mobile phase through the adsorbent bed is generally driven by capillary forces. It is much slower than in HPLC and its speed decreases even more with the distance from the mobile phase entry. This makes a TLC analysis relatively long and the separation distance quite limited. Another drawback of TLC is the fact that the separation is performed in a system that is not physiochemically equilibrated, and the separation conditions (e.g., mobile phase composition) may also depend on the distance from the mobile phase inlet. The separation system is opened to the gas phase; hence, the liquid components may undergo evaporation/condensation in various areas of the adsorbent layer.^1−3,7−9^

To overcome the main disadvantages of TLC, many attempts have been made to obtain forced flow of the mobile phase through the adsorbent layer in a fully closed separation system (deprived of contact with the gas phase). In general, there are two main ways to achieve the goal: the use of hydrodynamic flow (as in HPLC) or the use of the electroosmotic effect (EOF; analogically to capillary electrophoresis (CE) and capillary electrochromatography (CEC)).^1−3,9−16^ The former strategy is used in the already well-established technique: overpressured-layer chromatography (OPLC). In this technique, the adsorbent layer is covered by a flexible sheet (e.g., PTFE) and pressurized against its base/support. The use of a flexible cushion filled with a liquid (water) or gas under high pressure (usually 50 bar) provides uniform distribution of pressure over the whole adsorbent layer. The mobile phase is delivered to the adsorbent by the pump, with a pressure maximally as high as the pressure in the cushion. Commercially available chromatographic plates may be adapted to OPLC instruments by sealing their edges to prevent mobile phase leakage. The technique provides a rapid and uniform flow of the mobile phase through the adsorbent layer. This ensures fast and effective separations in various working modes. Two sample application modes, offline (to the dry chromatographic plate, as in TLC) or online (injection into the mobile phase flux, as in HPLC), can be combined with offline (the same as in TLC) or online detection modes (detector cell attached to the outlet capillary of the separation system). In the fully online mode, it is theoretically possible to perform separation in the physiochemically equilibrated system. All this makes OPLC an interesting and promising technique. However, most papers concerning OPLC focus rather on the offline application mode and normal-phase separation systems. In comparison, the online sample application seems to pose some technical difficulties and results in pronounced dispersion of solute zones/peaks.^1−3,9−12,17^

The other approach to force the mobile phase flow through the adsorbent layer is the use of EOF. It has some advantages, as it does not generate backpressure, as in the case of the hydrodynamic flow. Moreover, EOF is characterized by a flat profile, contrary to the laminar profile of hydrodynamic flow. This results in lower dispersion of solute zones during the separation. To be truly effective and to avoid some side effects, this approach also requires a completely closed separation system and a pressurized adsorbent layer, as in OPLC (achieved in a similar way with the use of the pressurizing cushion). Here, however, an electric potential is applied to the opposite edges of the adsorbent layer instead of pressure gradient. All this gave rise to development of another separation technique–pressurized planar electrochromatography (PPEC).^18−20^ This technique also facilitates fast separations carried out on long distances (in comparison to TLC). An additional advantage of the use of the electric field is the influence of the electrophoretic effect on the migration of ionizable solutes. Consequently, the separation selectivity of PPEC is different than that of TLC and OPLC. As in the case of OPLC, commercially available chromatographic plates can be adapted to PPEC by sealing their edges. Most papers on PPEC present offline sample application (with the exception of refs (21 and 22)) and, so far, only offline sample detection. This technique has been successfully used for separation of many various types of compounds, and its advantages make it an interesting alternative or supplement to the other separation techniques.^1−3,8^ However, as indicated in one of our latest papers, it has one serious drawback. As the mobile phase composition influences EOF, retention, and electrophoretic mobility of solutes at the same time, it is rather hard to optimize it to obtain satisfying results of separation of complex mixtures. The quite serious problem is that an attempt to avoid tailing of solute zones (e.g., by lowering pH of the mobile phase, addition of salts, buffers, ion-pairing reagents) very often leads to the reduction of EOF.^23^ It is worth mentioning that commercially available chromatographic plates with a silica-based nonpolar adsorbent layer provide extensive interactions of solutes with free silanols,^24,25^ contrary to HPLC adsorbents, which are designed to minimize such interactions. This only aggravates the problem.

A natural solution to the problems described seems to be combination of the advantages of both forced flow planar techniques OPLC and PPEC and overcome their limitations. In this context, the aim of our work is to present a design and construction of new prototype equipment combining both these techniques. Here we present the results of this work and some preliminary results of some separations performed with use of the new equipment.

## Experimental Section

### Chemicals and Equipment

Tartrazine (certified analytical standard) was purchased from the Institute of Dyes and Organic Products (Zgierz, Poland). Metafen and Poltram Combo Forte were purchased from Polpharma (Starogard Gdański, Poland). Sodium acetate (analytical purity grade) was purchased from Chempur (Piekary Śla̧skie, Poland). Methanol (for HPLC, super gradient) was purchased from POCH (Gliwice, Poland). Water used in all experiments was purified using an HLP demineralizer from Hydrolab (Gdańsk, Poland). Glass-backed HPTLC RP-18 W plates were purchased from Merck (Darmstadt, Germany).

### HPLEC Equipment

**Figure 1:**
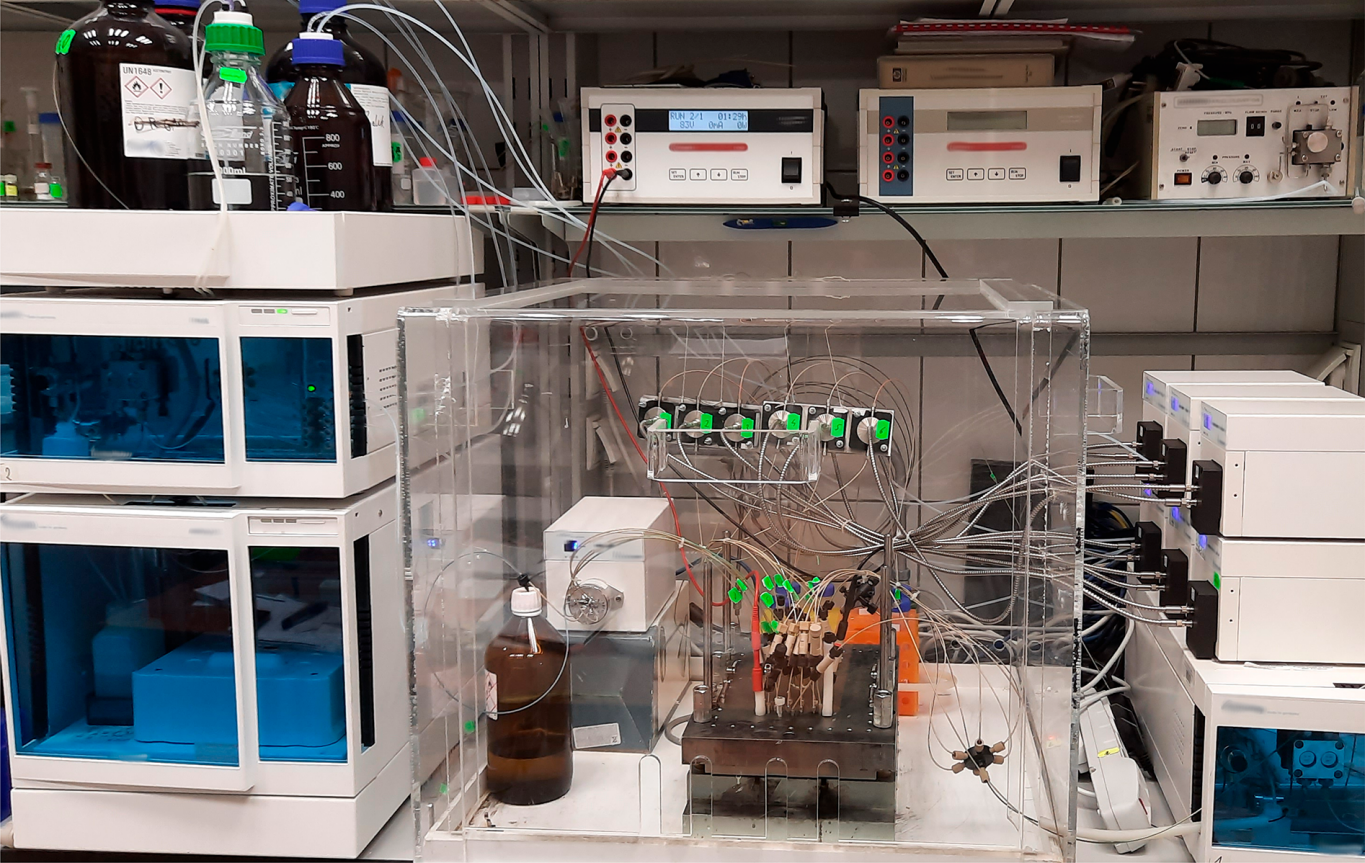
Prototype HPLEC chamber with coupled equipment.

**Figure 2:**
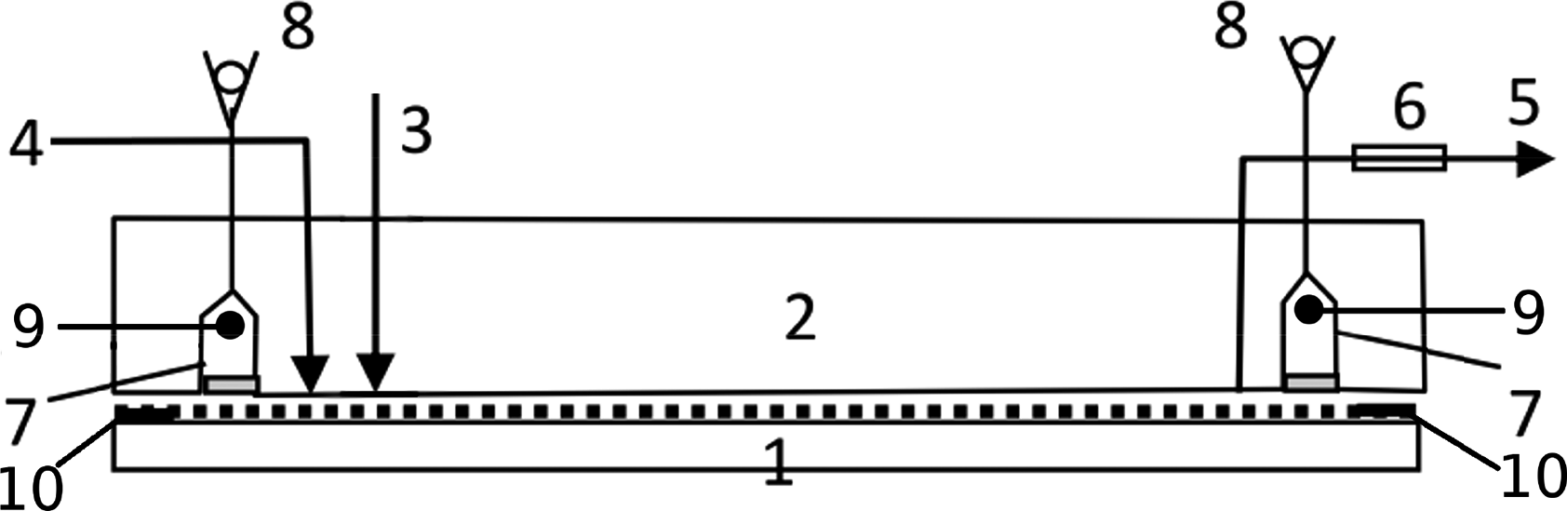
Conceptual scheme of the HPLEC separation chamber: 1, chromatographic plate with adsorbent layer face up sealed on its edges; 2, chromatographic plate cover with pressure cushion; 3, sample inlet; 4, mobile phase inlet; 5, mobile phase outlet; 6, detector cell; 7, electrode compartments; 8, rinsing/venting valves; 9, electrodes; 10, sealed margins of the chromatographic plate.

The prototype HPLEC device (Figure 1) has been designed and constructed in the Department of Physical Chemistry. Its main part is an electrochromatography chamber containing a chromatographic plate (10 cm × 20 cm) in which the separation process occurs. The conceptual scheme of the chamber is presented in Figure 2. The adsorbent layer on all edges of the plate is sealed with special silicone sealant using a 3D printer, making a 4 mm hermetic margin around the layer. The chromatographic plate is closed between two rigid steel elements: a chamber base body and a chamber cover body. During the separation, the base body and the cover body are connected with bolts and immobilized relative to each other. After removing the bolts, the cover is lifted up by springs, enabling insertion/removal of the chromatographic plate. The chamber base body contains a cooling pad connected to the circulating thermostat AD07R-20 (PolyScience, Niles, U.S.A.) and a thermocouple enabling temperature control during the separation process. The chromatographic plate is placed on the cooling pad with the adsorbent layer faced up. The chamber cover body contains a pressure cushion filled with a hydraulic liquid and connected to a special pressure supply unit purchased from P. W. Rafkop (Lubartów, Poland). The pressure supply unit is automatic and programmable; it provides constant pressure with the value required during the work of the HPLEC equipment. The pressure cushion pressurizes the chromatographic plate against the chamber base. The cushion is made of a chemically resistant flexible polymer, which adjusts itself to the adsorbent layer, providing uniform distribution of pressure over the whole adsorbent layer. Additionally, the cushion surface is covered with the PTFE layer to prevent adhesion of the adsorbent and increase the chemical resistance.

**Figure 3:**
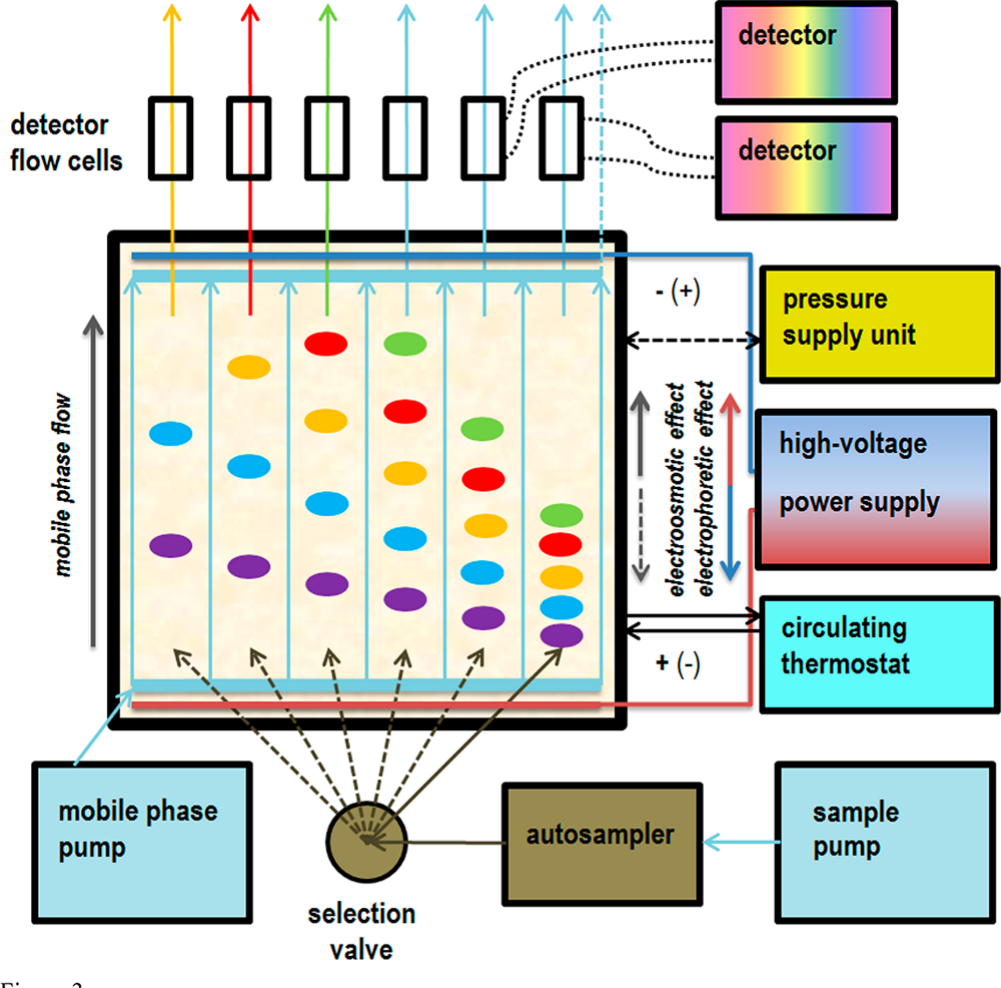
General scheme and conceptual principle of action of HPLEC equipment.

The pressure cushion contains two electrode compartments (5 mL volume) along the shorter edges of the chromatographic plate, placed 7 mm from its edge. Platinum wire electrodes connected to a high-voltage power supply EV262, Consort (Turnhout, Belgium), are placed and sealed in the electrode compartments located on the opposite sides of the cushion. Various inlet and outlet PEEK tubings are also sealed in the cushion. Two tubings are connected to each electrode compartment. Their tips extend close to the surface of the adsorbent layer enabling filling, drying, and rinsing the compartments, while the equipment is stopped. The third tubing (venting) is placed at the top of the compartments enabling removal of gas bubbles and/or rinsing the electrode compartments during the work of the equipment. The rinsing is performed by splitting the flow of the mobile phase delivered to the separation system using a valve/restrictor attached at the end of the venting tubing. This may occur by setting a constant flow (flow restrictor) or a constant pressure (back-pressure valve) to the venting capillary. Six inlet tubings delivering the mobile phase to the system are attached 17 mm from the shorter edges of the chromatographic plates. These are connected with a manifold to the quaternary HPLC pump (Azura P6.1L, Knauer, Berlin, Germany). The mobile phase is delivered to the trough, which is restricted from the electrode compartment to prevent mixing the “fresh” mobile phase delivered to the system with the mobile phase filling the compartments mentioned (the flow is directed from the trough to the compartment only). Six tubings delivering sample solutions (sample inlet) are placed 37 mm from the shorter edge of the plate. Another six tubings (outlet tubings) are placed 37 mm from the opposite edge of the plate. The 126 mm long adsorbent layer between the sample inlet and the sample outlet is the real separation distance. The sample inlet tubings are connected to an automatic six-channel selection valve (Azura V2.1S, Knauer, Berlin, Germany). The valve is connected to an autosampler (Azura AS6.1L) and a second quaternary HPLC pump (Azura P6.1L; both from Knauer, Berlin, Germany). The outlet tubings are connected to six separate analytical flow cells (path length 3 mm, capacity 2 μL). The flow cells are connected to six UV detectors (Azura UVD 2.1S, Knauer, Berlin, Germany). The general scheme of the HPLEC equipment and the conceptual principle of action are presented in Figure 3.

The electrodes combined to the high-voltage supply and the separation system (the mobile phase and adsorbent layer) are electrically insulated from any metal parts of the electrochromatography chamber, the pressure cushion, and all modules of the HPLEC equipment. For safety reasons, the metal elements of the chamber are additionally grounded. All potentially dangerous elements/modules of the equipment (in terms of high voltage) are closed in a special safety box during the system work (shown in Figure 1). Opening the safety box automatically disconnects the high-voltage power supply.

### Sample Preparation

For the test of sample application and detection, 5 mg/mL solution of tartrazine in the mixture of water/methanol (1/1 v/v) was used as a sample.

Metafen and Poltram Combo Forte solutions were prepared by dissolving a single tablet in 50 mL of methanol. The final concentrations of the drug in the Metafen solution (according to the composition declared by the producer) were 4 mg/mL of ibuprofen and 6.5 mg/mL of acetaminophen. The concentrations in the Poltram Forte Combo solution were 1.5 mg/mL of tramadol hydrochloride and 13 mg/mL of acetaminophen.

### OPLC and HPLEC Separation

The experiments were performed at the temperature of 25 °C and 100 bar pressure applied to the cushion pressurizing the adsorbent layer. A mixture of water/methanol (2/3 v/v) with addition of 50 mM sodium acetate (final concentration) was the mobile phase. Most of the HPLEC equipment modules were controlled by a computer with ClarityChrom software (Knauer, Berlin, Germany). Only the high-voltage power supply, the pressure supply, and the circulating thermostat were programmed independently.

Before separation, the adsorbent layer was washed (conditioned) with the mobile phase for 1 h. For the HPLEC experiments, voltage gradient from 0 V to the final 2 kV was applied at the same time. Using an autosampler, 1 μL of the sample solution was injected into the stream of the mobile phase pumped by the sample pump. Then, the sample solution in the stream of the mobile phase was delivered to the virtual channel of the chromatographic plate. The ratio of the flow speed from the sample pump to the total flow of the mobile phase was 15%. The flow from the sample pump was applied sequentially to one channel for 3 min and then switched to the next channel; hence, there was a 3 min delay in sample injection between the subsequent channels. After injection of the samples to the 5 channels, the mobile phase pumped by the sample pump was switched to the waste and the sample pump was stopped. Only 5 of the 6 channels were used (nos. 1–5), as the position no. 6 of the selection valve was used to switch the flow from the sample pump to the waste before and after the sample injection.

During the separation, the venting valve of the inlet side electrode compartment was opened to facilitate constant rinsing of the electrode with the mobile phase. The flow through the venting valve was restricted to 2% of the total mobile phase flow. On the outlet side, the flow was 7% for each detector cell and 56% for the electrode venting valve. No voltage was used (OPLC mode) for the tartrazine application/detection test. The total flow of the mobile phase was 0.25 mL/min and the backpressure was 86 bar. The separation of the Metafen and Poltram Combo Forte solutions was performed at 2 kV, total flow of 0.30 mL/min, and backpressure of 83 bar.

Tartrazine was detected at 256 nm, while acetaminophen, ibuprofen, and tramadol, at the 210 nm wavelength, simultaneously using six independent flow cells and detectors, each collecting samples/eluents from six independent separation channels of the HPLEC chamber. Six independent signals were recorded and overlaid in a single analysis (chosen signals may be shown/hidden at any time).

## Results and Discussion

As the main result of our work, we present completely new analytical equipment designed and constructed in our laboratory. The equipment was designed to mix the advantages and overcome the limitations of two planar separation techniques known so far: OPLC and PPEC. For the combination of these two, we propose a draft name: high-performance (high-pressure) layer electrochromatography (HPLEC). The equipment described can be used for OPLC or PPEC separations (the latter at least in the offline detection mode, as the possibility of online detection is not certain and needs to be confirmed). It allows both offline and online sample application. Similarly, offline and online solute detection is possible. The equipment is meant to give a possibility of simultaneous analysis of multiple samples in a single analysis. Constant or temporary flow from the sample pump may be used, as well as various methods of online sample application, depending on the ratio between the solvent flow from the main pump and the sample pump. Free manipulation of electrical voltage is possible as well (switching on/off at any time, setting the required value, using voltage gradient, etc.). Most importantly, the flow of the mobile phase is independent of the electroosmotic effect. The equipment is also designed to use a gradient of the mobile phase. Pressure of the mobile phase up to 100 bar can be used. The separation temperature can be controlled. As the sample is applied with the use of a second independent pump, it can be injected in the stream of the mobile phase with different compositions/elution strengths. The electrode compartments can be rinsed with the mobile phase, thereby facilitating the removal of products of electrode reactions and preventing their influence on the separation process. The flow of the rinse and the flow of the solutes/mobile phase reaching the detector cells can be controlled. Some of these theoretical/constructional features of our equipment can be confirmed by our preliminary results of the analysis presented below.

**Figure 4:**
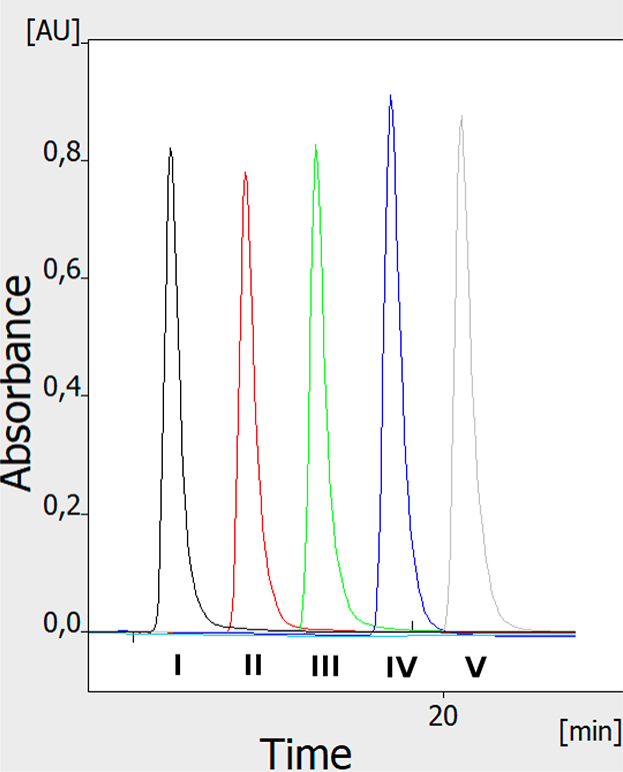
Fully online multichannel test of sample application and detection (OPLC mode). A total of 1 μL of tartrazine (5 mg/mL) was applied to the five separation channels (I–V). The delay between the sample application in each subsequent channel was 3 min. Separation system used: HPTLC RP-18 W chromatographic plates; water/methanol (2/3 v/v) with the addition of 50 mM sodium acetate as the mobile phase; total mobile phase flow 0.25 mL/min; voltage 0 kV; temperature 25 °C, cushion pressure 100 bar; mobile phase backpressure 86 bar.

To investigate whether the profile of the mobile phase flow through the adsorbent layer is smooth and uniform, we performed a multichannel test of sample application and detection. Figure 4 shows an OPLC chromatogram of tartrazine injected sequentially (3 min between subsequent injections) into the five channels of the separation system. Since the distances between all subsequent peaks on the chromatogram are the same, the flow of the mobile phase and the solutes is proved to be uniform throughout the whole adsorbent layer. Our results (not shown here) concerning the response of detectors to the mobile phase front during the adsorbent prewetting and initial gradient separation attempts also support that claim. Moreover, the shape of all peaks is relatively similar, as well as their height and area. This proves that the fully online application and detection of the sample is quite repeatable for all the five separation channels (the sixth channel was not used, as the sixth position of the selection valve was set to the waste). It is worth mentioning that the individual detectors provide an equivalent response; it was examined by testing detectors without HPLEC chamber connected (results not shown).

**Figure 5:**
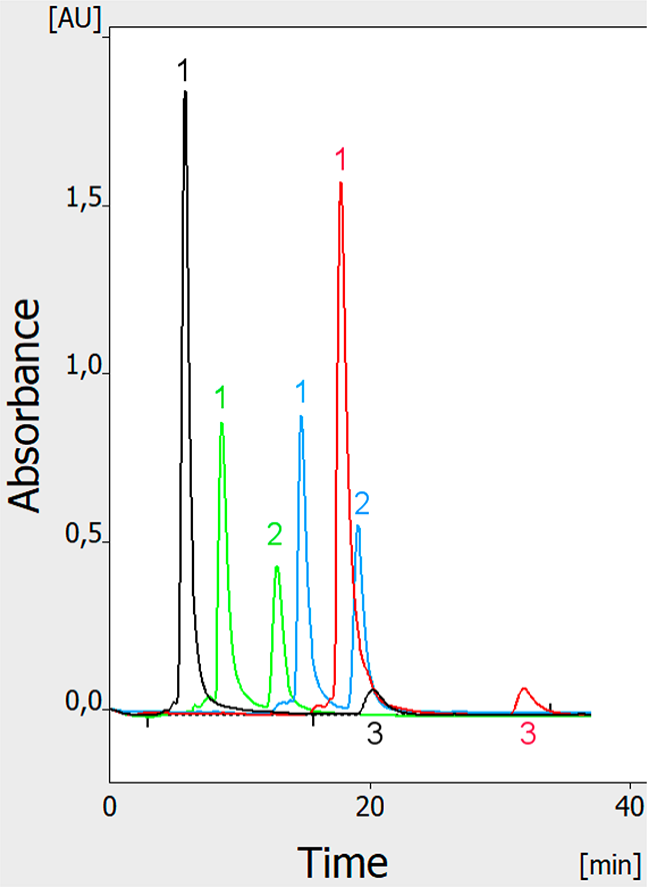
Fully online multichannel HPLEC of Poltram Combo Forte, black and red (channel nos. 1 and 5, respectively), and Metafen, green and blue (channel nos. 2 and 4, respectively). Channel no. 3 is hidden. A total of 1 μL of the drug solution was applied to the five separation channels. The delay between the sample application in each subsequent channel was 3 min. Separation system used: HPTLC RP-18 W chromatographic plates; water/methanol (2/3 v/v) with addition of 50 mM sodium acetate as the mobile phase; total mobile phase flow 0.3 mL/min; voltage 2 kV; temperature 25 °C, cushion pressure 100 bar; mobile phase backpressure 83 bar. Detected solutes: 1, acetaminophen; 2, ibuprofen; 3, tramadol.

To demonstrate the practical application of our equipment, we performed simultaneous multichannel HPLEC analysis of solutions of two different analgesic drugs: Metafen and Poltram Combo Forte (Figure 5). Only four of the five electrochromatograms obtained simultaneously are presented, as that from channel no. 3 is hidden to improve the clarity/readability of the figure. The results prove that our equipment allows performing multiple separations at the same time, increasing the throughput of the analysis. Additionally, a set of solute standards can also be injected in parallel with the sample analyzed. The proper migration time of solutes must be calculated taking into account the real injection time, as the current software does not make this correction automatically. Moreover, some minor differences in the peak shape and height/area can be noticed. They are probably related to the precision of the elaboration of some “hand-made” HPLEC chamber elements (such as flexible membrane pressurizing the adsorbent, PTFE sheet, especially at inlets and outlets of the sample). Also, many different junctions can cause some problems, the same as deformation (and repeatability of technical parameters) of PEEK tubings. Surely these elements need further improvement and require some professional engineering and production technology.

On the other hand, optimization of sample application and “collection” (direction to the flow cell) may be crucial for the detection. This includes setting of the proper ratio between mobile phase and sample flow, as well, as proper splitting of the sample between detection cells and outlet-side electrode compartment. Moreover, the part of the sample directed to the detection cells can possibly be additionally affected by the electric voltage (due to electrophoretic effect). These issues require further detailed investigation and discussion.

In comparison to PPEC, the equipment presented here allows obtaining the required flow of the mobile phase irrespective of the voltage used and the mobile phase composition. The flow is forced by the pump, not by the electroosmotic effect; therefore, the composition of the mobile phase can be freely optimized to obtain the retention needed. Also, the voltage can be freely optimized to obtain the electrophoretic effect required and further changes in separation selectivity. This is the advantage in comparison to OPLC. Our results show that another advantage of HPLEC is the possibility of use of the electroosmotic effect to additionally facilitate the flow of the mobile phase and reduce backpressure at the inlet of the separation chamber. The backpressure in the OPLC mode was 96 bar at the mobile phase flow of 0.25 mL/min, while with use of voltage (HPLEC mode) it was even lower (93 bar) at the higher flow (0.3 mL/min) in a similar separation system. Therefore, the use of voltage may facilitate the application of higher flow velocities of the mobile phase in comparison to OPLC. Theoretically, our equipment also allows performing separation against the electroosmotic effect if it can be predominated by the hydrodynamic flow. This, however, needs to be proved and requires further research and investigations. Nevertheless, the presented technique offers more different possibilities of analysis and ways of system optimization than any other separation technique presented so far.

The HPLEC system seems to be relatively susceptible to scaling: all that is needed is a change in the dimensions of the separation chamber and the number of separation channels. The limitations are in fact technical capabilities. The equipment presented here is only the first HPLEC prototype, and very preliminary results are described. Our aim, however, is to indicate the potential of this analytical technique. Considering the precision and resolution of modern ink printers, it is easy to imagine HPLEC equipment working with similar or at least close parameters (resolution, precision and accuracy), as both use a flat (planar) medium along with liquids. Just proper technological solutions are required, but professional engineering remains out of our reach, at least for the time being. Naturally, there are also some problems with the online detection, as multiple separation channels require multiple detectors. However, some multichannel detectors may probably be produced (e.g., with the use of diode arrays). Another solution may involve the use of a selection valve and sequential redirection of samples from multiple separation channels to a single detector (at the cost of final sampling frequency). The possibility of finding some other, maybe quite different, solutions must be taken into account.

## Conclusions

The HPLEC technique proposed here offers a wide range of variables/parameters that can be easily optimized to obtain the best separation. It combines the advantages and overcomes the limitations of both OPLC and PPEC. The prototype equipment presented here can work in many different modes of the mechanism of separation (OPLC, PPEC, HPLEC) and with various combinations of sample application and detection (online/offline). Most importantly, it provides simultaneous multichannel, fully online, and fully automated high-throughput analysis. With application of modern advanced technological solutions, proper engineering and precise manufacturing, scaling, and minimization, HPLEC might reveal its full capabilities and become competitive in the field of analytical science and industry.
